# Transposable element abundance correlates with mode of transmission in microsporidian parasites

**DOI:** 10.1186/s13100-020-00218-8

**Published:** 2020-06-23

**Authors:** Nathalia Rammé Medeiros de Albuquerque, Dieter Ebert, Karen Luisa Haag

**Affiliations:** 1 Department of Genetics and Post-Graduation Program of Genetics and Molecular Biology, Federal University of Rio Grande do Sul, Av. Bento Gonçalves 9500, Porto Alegre, RS 91501-970 Brazil; 2 Department of Environmental Sciences, Zoology, Basel University, Vesalgasse 1, 4051 Basel, Switzerland

## Abstract

The extreme genome reduction and physiological simplicity of some microsporidia has been attributed to their intracellular, obligate parasitic lifestyle. Although not all microsporidian genomes are small (size range from about 2 to 50 MB), it is suggested that the size of their genomes has been streamlined by natural selection. We explore the hypothesis that vertical transmission in microsporidia produces population bottlenecks, and thus reduces the effectiveness of natural selection. Here we compare the transposable element (TE) content of 47 microsporidian genomes, and show that genome size is positively correlated with the amount of TEs, and that species that experience vertical transmission have larger genomes with higher proportion of TEs. Our findings are consistent with earlier studies inferring that nonadaptive processes play an important role in microsporidian evolution.

## Background

Microsporidia, a phylum of fast evolving intracellular parasites related to fungi [1–3], are interesting models for studying the mechanisms shaping eukaryotic genome evolution. Their genomes are small and highly diverse. Two contrasting genome architectures are found in microsporidians. The extremely compact, gene-dense genomes of the encephalitozoonids (≅ 2 Mb), which include human parasites, are the smallest known eukaryote parasites [4, 5]. At the other extreme are the large, gene-sparse genomes of several phylogenetically distant species, such as *Edhazardia aedis* (≅ 50 Mb), *Hamiltosporidium* spp. (≅ 17–25 Mb), *Nosema bombycis* (≅ 15 Mb) and *Anncaliia algerae* (≅ 12–17 Mb) [6–10]. Despite showing an about 25-fold difference in size, the gene-sparse and gene-dense genomes only differ by a factor of 2–3 in the number of proteins they encode, and a large fraction of the noncoding regions in the gene-sparse genomes is populated by repetitive sequences, most of which have been identified as transposable elements [11]. It is suggested that the majority of transposable element (TE) insertions cause mildly deleterious effects in eukaryotes [12, 13]. Therefore, their spread and accumulation depend on the cellular mechanisms of TE silencing [14] as well as on the efficacy of natural selection to eliminate or retain them [15]. TEs are also known to escape cellular defense mechanisms by transferring themselves horizontally to different genomes [16, 17]. When a TE enters a genome for the first time, it can often duplicate freely before becoming epigenetically silenced [14]. Symbiotic organisms living in close proximity are more likely to exchange TEs [16, 18]; not surprisingly, a large number of horizontal gene transfer (HGT) events involving TEs have been identified in the evolutionary history of microsporidia [11].

We have recently proposed that nonadaptive processes have a major role in the evolution of microsporidian species that are predominantly vertically transmitted [8]. In such species genetic drift might override selection because the translocation of parasites from the host somatic cells to gametes reduces the parasite effective population size (*N*_*e*_) [19]. Moreover, vertical transmission might be predominant in host species with a metapopulation structure, where local extinction and re-colonization is common and where the parasite enters new populations with the hosts dispersal stage and thus experiences severe bottlenecks [20].

Natural selection is efficient only if selection coefficients are greater than about 1/2*N*_*e*_ [21]. In our previous study we showed that vertical transmission in a subset of microsporidian genomes is associated with a higher ratio of non-synonymous to synonymous substitutions [8]. Furthermore, microsporidia that experience vertical transmission usually have gene-sparse genomes [8]. Here we expand our analyses by characterizing the mobilomes from a larger number of genomes, and explore TE evolutionary dynamics in the light of microsporidian modes of transmission.

## Results and discussion

**Fig. 1:**
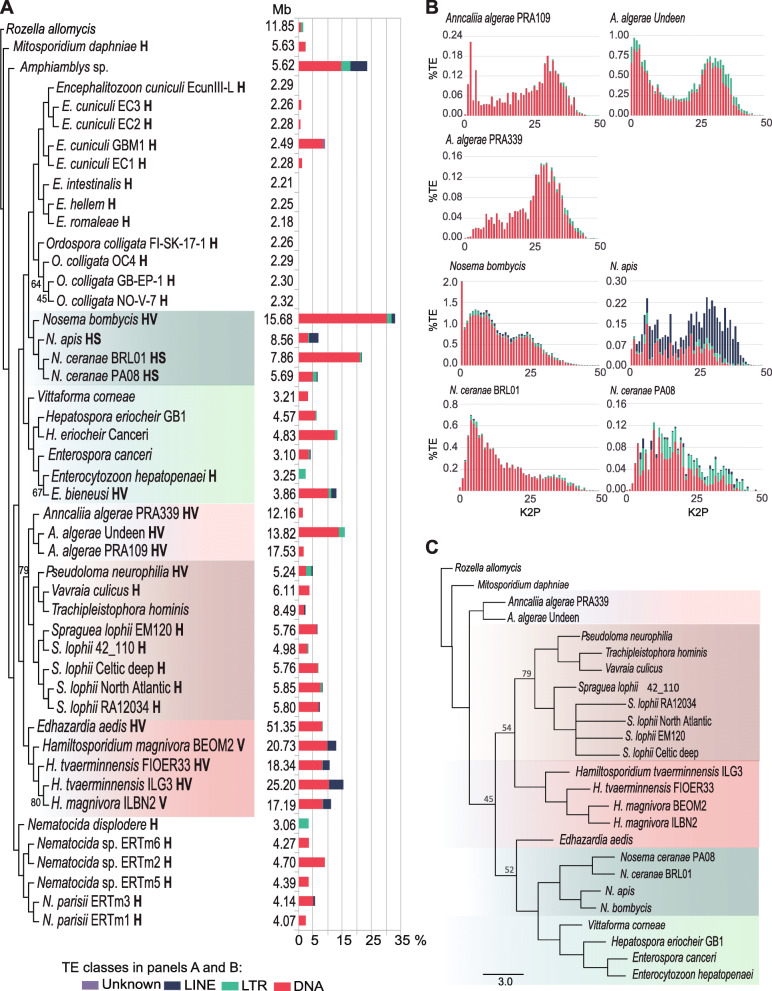
TE dynamics in the history of microsporidia. a Assembly sizes (Mb) and their percentages of distinct TE classes are displayed next to the phylogeny of 47 microsporidia. The tree was inferred using 37 highly conserved proteins; only bootstrap support values lower than 90% are shown. Known modes of transmission are indicated in bold letters: H = horizontal; HV = horizontal and vertical; HS=Horizontal sexual. b Age distribution of different classes of TEs; larger Kimura distances (K2P) represent older elements. c Phylogeny inferred using concatenated Argonaute and Dicer proteins from 25 lineages of microsporidia. Only lineages where both proteins were found have been included in the analysis. Rozella allomycis, an early diverging fungus [3], is used as root in both (a) and (b) phylogenies⁠. The major clades of microsporidia bearing both Argonaute and Dicer are highlighted in different colors

All 47 microsporidian genome assemblies available as of March 2019 were retrieved from GenBank (Additional file 1) and used for the identification of TEs. DNA transposons are the dominant class of TEs in microsporidian genomes, but their abundance varies greatly between species, and is not phylogenetically conserved (Fig. 1a; Additional file 2). The tree presented in Fig. 1a, built from a dataset of 37 highly conserved proteins (Additional file 3) established to reduce long-branch-attraction artifacts in microsporidian phylogenies [1, 2], is congruent with previously published microsporidian phylogenetic inferences based on other data [22, 23].

To evaluate whether TE accumulation in microsporidian lineages represents ancient or recent events, we used the Kimura 2-parameter distance (K2P) to generate age distributions of TEs within each genome (Additional file 4). The evolutionary dynamics of TEs shows very distinct patterns, even within species. For example, the three *A. algerae* genomes show variable amounts of TE content (1.64% in strain PRA109, 1.34% in PRA339 and 15.75% in Undeen; Additional file 1; Fig. 1a), and the accumulation of TEs in *A. algerae* Undeen apparently results from a recent spread of DNA transposons, mainly from the Merlin superfamily (Additional Files 5 and 6). A sharply bimodal distribution of TE abundance per age class suggests two successive expansions in *A. algerae* Undeen that are not observed in the remaining two strains (Fig. 1b). Similarly, the *Nosema* genomes from *N. bombycis* (33.41% of TEs) and *N. ceranae* BRL01 (21.64% of TEs) show an excess of young elements, which also seems to result from the recent spread of Merlin (Fig. 1b; Additional Files 5 and 6).

We reasoned that these recent events of TE expansion in some evolutionary lineages could result from the loss of genes encoding components from cellular defense mechanisms, such as members of the RNAi pathway. We test this idea using the example of *Dicer* and *Argonaute*, two genes essential for the RNAi pathway. It was suggested that *Dicer* and *Argonaute* genes are selected as a unit in microsporidia, and that they were selectively maintained or lost during lineage divergence [24]. Therefore, we searched for orthologs of proteins related to cellular defense against TEs in the 47 proteomes (Additional file 6). No RIP/MIP-related proteins, which silence TEs by inducing mutation and/or cytosine methylation within repetitive sequences, were found in any lineage. Most strains carrying *Dicer* and *Argonaute* orthologs also carry the protein sets necessary for somatic quelling and meiotic silencing, but this is still no evidence of the activity of these mechanisms in microsporidia. The number of *Dicer* and *Argonaute* gene copies varies between 0 and 5 and 0–7, respectively, and in one lineage (*A. algerae* PRA 109) we found 2 copies of *Dicer*, but no *Argonaute* (Additional file 6). Thus, *Dicer* and *Argonaute* seem not necessarily selected as a unit. Interestingly, although there is an overall congruence between the *Dicer* + *Argonaute* tree (Fig. 1c) and the one built with 37 conserved proteins (Fig. 1a), there are two inconsistencies, i.e., the *A. algerae*, and the *E. aedis* clades, though with low statistical support.

**Fig. 2:**
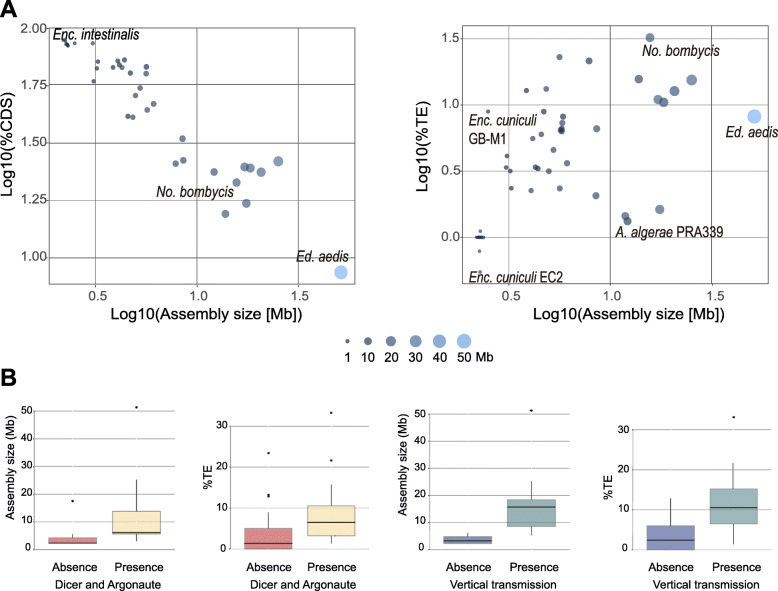
TE accumulation associated with parasite vertical transmission. a Spearman correlations of assembly sizes (Mb) with their coding (CDS) and TE fractions. b Distributions of assembly sizes and TE fractions in lineages grouped according to the presence or absence of both Argonaute and Dicer, and to the presence or absence of vertical transmission.

A strong negative correlation was found between the percentage of total coding sequences (CDS; including both TE and host derived sequences) and genome size (rho = − 0.92, *p*-value = 2.2e-16; Fig. 2a), indicating that non-coding regions are responsible for variations in genome size*.* On the other hand, there is a positive correlation between the fraction of genomes occupied by TEs and genome size (rho = 0.66, *p*-value = 4.375e-07; Fig. 2a). Regression analysis between TE fraction and genome size is also significant (R-squared = 0.33, *p*-value = 1.501e-05; Additional file 7). We further applied phylogenetic independent contrasts to the regression analysis [25] in order to correct for the non-independency of traits among taxa, which showed that the positive correlation between genome size and the amount of TEs in microsporidia does not result exclusively from phylogenetic inertia (R-squared = 0.16, *p*-value = 0.003; Additional file 7). However, some lineages with comparatively large genomes such as *A. algerae* PRA 109, PRA339 and *Edhazardia aedis* have TE fractions similar to small genomes such as *Encephalitozoon cuniculi* GBM1 and *Enterocytozoon bieneusi.* Both *E. cuniculi* GBM1 and *E. bieneusi* genomes suggest recent expansions of miniature inverted-repeat transposable elements according to the K2P age distributions of TEs (MITEs; Additional files 3 and 5)*.* MITEs are deletion derivatives of DNA transposons from the *Tc1/Mariner* superfamily [26]. *Tc1/Mariner* elements are absent in *Encephalitozoon cuniculi* and *Enterocytozoon bieneusi* but are found in closely related microsporidia (*Nosema* spp. and *Hepatospora eriocheir*; Additional files 3 and 5).

We identified Dicer and Argonaute orthologs in 27 proteomes (Additional file 6). Strains with putative *Dicer* and *Argonaute* genes have, on average, a higher proportion of TEs (8.2%), and larger genome sizes (11.05 Mb) compared to strains in which these proteins are absent (4.06% and 3.81 Mb, respectively; Wilcoxon rank sum; *p*-value < 0.004, and p-value < 2.2e-16, respectively; Fig. 2b). Although the association between the presence of both *Dicer* and *Argonaute* with the accumulation of TEs seems to support the idea that a RNAi machinery is being selectively maintained in microsporidian genomes attacked by selfish elements, we think that random events may have had an equivalent or perhaps more important role. First, there is only partial phylogenetic congruence between *Dicer* + *Argonaute* proteins (Fig. 1c) and the history of microsporidia inferred from 37 highly conserved proteins (Fig. 1a), suggesting rate heterogeneity in the evolution of the microsporidian RNAi machinery. Second, genes involved in the RNAi pathway may have been lost by deletion or gained by duplications and/or HGT. For example, although it cannot be ruled out that genes might be lacking in the assemblies due to genome incompleteness, and that microsporidia may have mechanisms to eliminate TEs other than RNAi, *Argonaute* was apparently lost in *A. algerae* PRA109 but not in *A. algerae* PRA339, in spite of both strains having a similar TE age structure (Fig. 1b). We have also found evidence suggestive of *Argonaute* HGT involving *N. bombycis*; its Argonaute protein sequence is identical to *Papilio xuthus,* the Asian swallowtail butterfly (Additional file 8). The ortholog protein in *N. apis* has only 62.3% identity with the *P. xuthus* Argonaute. Previous comparative studies focusing on the enlargement of the *N. bombycis* genome suggested a major implication of HGT in the acquisition of new genes and TEs from its lepidopteran host *Bombyx mori* [10]. However, in the particular case of *Argonaute*, HGT may have occurred in the opposite direction. The genome of the butterfly *Papilio xuthus* encodes orthologes of Argonaute that cluster with microsporidian proteins (Additional file 8). Two out of three *P. xuthus* microsporidian-like *Argonaute* genes are found on large contigs (NW_013531057.1, length = 195,660 bp; and NW_013530778.1, length = 550,741 bp), weakening the hypothesis that they represent assembly contamination with microsporidian DNA.

Horizontal transfer of different genes has been reported in microsporidia [23, 27–30]. Among TEs, DNA transposons are the most often horizontally transferred in eukaryotes [16], and in *A. algerae,* several *Merlin* and *PiggyBac* transposon insertions were shown to result from HGT [11]. We hypothesize that the invasion and accumulation of TEs in microsporidian genomes is facilitated by their intracellular mode of life and by the low power of purifying selection. We further hypothesize that the latter is caused by low *N*_*e*_ in association with vertical transmission. Microsporidia use vertical transmission as a survival strategy when opportunities to transmit horizontally are reduced [31], thus being of particular importance during times of low density or the colonization of new host population, both of which cause population bottlenecks. Therefore, this mode of transmission may reduce *N*_*e*_ and thus the strength of natural selection [8]. Species with larger mobilomes, such as *N. bombycis* and *Hamiltosporidium* spp. show mixed mode transmission (a combination of horizontal and vertical transmission) [32, 33]. Microsporidia with mixed-mode transmission have, on average, a higher proportion of TEs than species with horizontal transmission. The difference in TE percentage between species capable of vertical transmission (11.48%) and those showing only horizontal transmission (3.37%) is statistically significant (Wilcoxon rank sum test, *p*-value < 0.0006; Fig. 2b). The difference in genome size between these two groups is also significant (Wilcoxon rank sum test, *p*-value = 1.77e-05; Fig. 2b), with vertically transmitted species having larger genome sizes, on average (16.87 Mb, against 3.67 Mb, from species exclusively horizontally transmitted).

On closer inspection, one can see that some microsporidia that use vertical transmission do exhibit a low proportion of TEs (such as *A. algerae* PRA109, PRA339 and *E. aedis*; Fig. 1; Additional files 2 and 4). While these examples are few and do not reverse the overall statistical signal, they seemingly contradict our hypothesis. It is unclear if these low abundances are genuine or artefacts resulting from current methodological approaches. TEs are a challenge for genome assembly and annotation, and some sequencing pipelines may discard TEs during the assembly process and/or later during the filtering of contaminants [34]. Different sequencing platforms and pipelines were used to sequence and assemble the genomes used in our study, which might have interfered with subsequent TE annotation. Most TE annotation methods rely on homology-based approaches that limit the discovery of novel families of TEs, or require previous knowledge of structural features of TEs, being prone to false negatives [35]. Moreover, highly degenerated TEs without recognizable structural features can be missed from annotation [36, 37].

Taken together with our previous study [8] the here presented results show that vertical transmission is correlated with genome expansions and the spread of TEs in microsporidia. Thus, we suggest that nonadaptive forces play a strong role in shaping the evolution of genome size in populations of microsporidian parasites [13].

## Materials and methods

### TE annotation

For de novo identification of TE in all genomes we used RepeatModeler software with default parameters. RepeatModeler creates consensus sequences for each TE family and classifies based in homology. The output is TE lineage-specific libraries for each genome. We combined these TE lineage-specific libraries with all TE from fungi obtained from Repbase to be used in RepeatMasker software [38, 39]. RepeatMasker is a homology-based method that screens genomic sequences and annotates TEs by using BLAST (e-value threshold of 1e-10) as search engine. Low complexity DNA sequences and simple repeats were not masked. The annotation of Miniature Inverted-Repeat Transposable Elements (MITEs) insertions was performed with MITE Tracker [40]⁠. The software identifies elements with valid Terminal Inverted Repeats (TIRs) between 50 and 800 nt, and Target Site Duplications (TSDs). MITE candidates are filtered by flanking sequence (sequences outside the TSDs with 50 nt in length) similarity and grouped into families. MITEs undetected or classified as “unknown” by RepeatModeler were added or reclassified in the TE lineage-specific libraries. The tool “One code to find them all” [41]⁠ was used to filtrate retrotransposons longer than 80 bp and DNA transposons longer than 50 bp, with > 80% of identity with the reference sequences. The output summarizes the number of TE copies and genome coverage for each TE family.

### Kimura distance and genome coverage

The Kimura 2-Parameter (K2P) divergence metric was calculated for TE sequences present in all genomes analyzed with RepeatMasker package RepeatLandscape [39, 42]. RepeatLandscape creates graphs depicting the contribution of repeat classes for each genome assembly and K2P distance from the consensus TE sequences contained in the RepeatMasker libraries.

### Highly conserved proteins for phylogenetic analyses

We searched the 47 genome assemblies for highly conserved proteins [1, 2] using BLASTp (e-value threshold of 1e-15). The conserved proteins needed to be present in at least half of the analyzed proteomes for not being removed in the multiple alignment trimming phase. We identified 37 conserved proteins in at least 50% of the proteomes (Additional file 3).

### Genome defense mechanisms against TEs

We searched for proteins related to genome defense mechanisms against TE [43]. BLASTp searches with e-value threshold of 1e-5 were performed for using *N. crassa* and other ascomycete proteins as queries: RNA-dependent RNA polymerase (Q1K6C4), DNA repair protein RAD51 (Q1K929), DNA repair protein RAD52 (Q9HGI2), DNA repair protein Rad54 (Q9P978), Replication protein A (Q1K7R9), RNA helicase (Q7S3A3), Masc1 (Methyltransferase from Ascobolus 1) (O13369), RID (RIP defective) (V5INW3). BLASTp searches with e-value threshold of 1e-8 were performed using *Hamiltosporidium tvaerminnensis* FI-OER-3-3 Dicer (A0A4Q9LBM7) and Argonaute (A0A4Q9L0B4) as queries (Additional file 6).

### Phylogenies

Protein or nucleotide sequences were aligned with MUSCLE and uninformative columns in the alignments, containing at least 50% of gaps, were trimmed using Geneious Prime 2019.0.4 [44, 45]. The best amino acid substitution models fitting our data were selected with ProtTest 3.4.2 [46]. The 37 conserved proteins were concatenated, and the final alignment contained 12,467 sites. The best model according with the AIC and BIC criteria was VT + I + G. Argonaute and Dicer proteins were concatenated and the final alignment contained 1822 sites. The best model was LG + I + G. In order to investigate the direction of Argonaute HGT, we selected and aligned representative nucleotide sequences from *Nosema* spp. and Lepidoptera. The best model according with the AIC criteria was GTR + G. The maximum likelihood phylogenies were estimated using in RAxML 8.2.10 [47], and the statistical support for each branch was estimated by 1000 bootstrap replicates.

### Statistical tests

In order to test correlations between TE content and genome size all data were logarithmically transformed. We applied Shapiro-Wilks method [48] to test the normality of our data with the function shapiro.test() in R version 3.6.2. The data were not normally distributed therefore nonparametric tests were applied. The Spearman correlation between TE percentage in the genome and genome size, as well as total CDS length and genome size, was determined by using the function cor.test() in R [49]. The package ape in R [50] was used to perform a regression analysis between the amount of TEs and genome size using the function fit.pic(). We performed a Wilcoxon rank sum test with the function wilcox.test() in R to test for a difference in TE percentage between lineages horizontally transmitted vs. lineages with mixed-mode transmission, and genome size between lineages horizontally transmitted and lineages with mixed-mode transmission (Additional file 9). We also tested the difference in TE percentage between genomes encoding for both Dicer and Argonaute proteins vs. genomes where these proteins are absent. The plots were constructed using ggplot2 package in R [51].

## Supplementary information

**Additional file 1.** Microsporidian assemblies analyzed in our study.

**Additional file 2.** Content of distinct TE classes in the microsporidian assemblies.

**Additional file 3.** List of the 37 highly conserved proteins employed for phylogenetic reconstruction.

**Additional file 4.** Distribution of the Kimura 2-Parameter (K2P) divergence metric calculated for all TE sequences present in all genomes analyzed in our study.

**Additional file 5.** Frequency of subtypes of TEs in microsporidian mobilomes.

**Additional file 6.** Results of BLASTp searches for proteins involved in genome defense mechanisms against TEs.

**Additional file 7.** Regression analyses of the amount of TEs in relation to genome size with and without phylogenetic independent contrasts.

**Additional file 8.** Protein alignment of representative microsporidian and lepidopteran Argonaute sequences; Argonaute phylogeny testing the direction of the HGT event.

**Additional file 9.** Known transmission modes of the microsporidia included in our study.

## Data Availability

The datasets generated as part of the study are available as supplementary information. Please contact the authors for further data request.

## Abbreviations

HGT: Horizontal Gene Transfer
K2P: Kimura 2-Parameter distance
RIP: Repeat-induced Point mutation
MIP: Methylation Induced Premeiotically
MITE: Miniature Inverted-repeat Transposable Element
TIR: Terminal Inverted Repeat
TSD: Target Site Duplication
CDS: Coding sequence
AIC: Akaike Information Criterion
BIC: Bayesian Information Criterion
