# Supplementary material for: Transposable element abundance correlates with mode of transmission in microsporidian parasites
Source: Mob DNA. 2020 Jun 23;11:19. doi: 10.1186/s13100-020-00218-8 (PMC7313128; doi:10.1186/s13100-020-00218-8)
Supplement: Supplementary file 4 — Additional file 4. Distribution of the Kimura 2-Parameter (K2P) divergence metric calculated for all TE sequences present in all genomes analyzed in our study. [file 13100_2020_218_MOESM4_ESM.pdf]

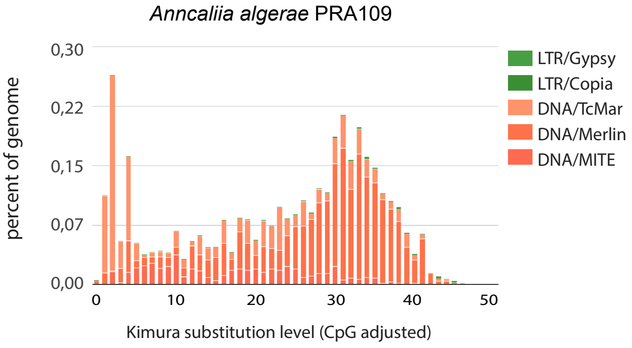
Anncaliia algerae PRA109
percent of genome
0,30
0,22
0,15
0,07
0,00
0
10
20
30
40
50
Kimura substitution level (CpG adjusted)
LTR/Gypsy
LTR/Copia
DNA/TcMar
DNA/Merlin
DNA/MITE

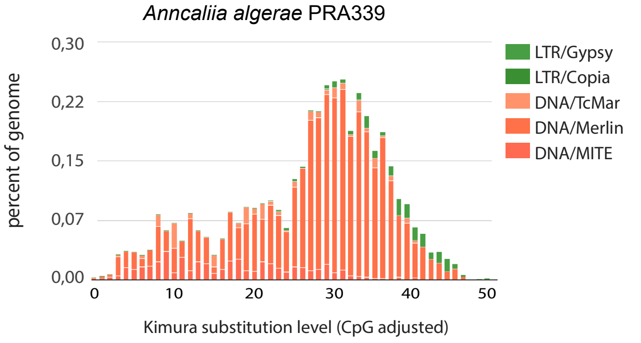
Anncaliia algerae PRA339
percent of genome
0,30
0,22
0,15
0,07
0,00
0
10
20
30
40
50
Kimura substitution level (CpG adjusted)
LTR/Gypsy
LTR/Copia
DNA/TcMar
DNA/Merlin
DNA/MITE

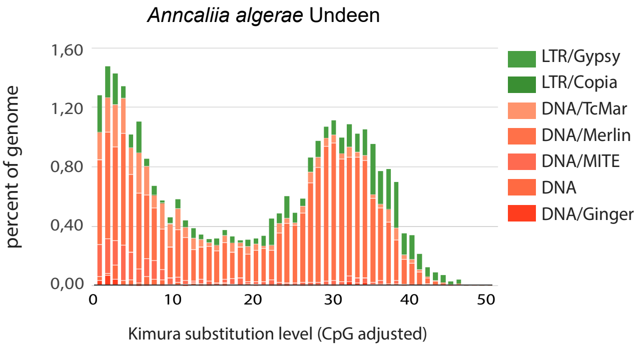
Anncaliia algerae Undeen
percent of genome
1,60
1,20
0,80
0,40
0,00
0
10
20
30
40
50
Kimura substitution level (CpG adjusted)
LTR/Gypsy
LTR/Copia
DNA/TcMar
DNA/Merlin
DNA/MITE
DNA
DNA/Ginger

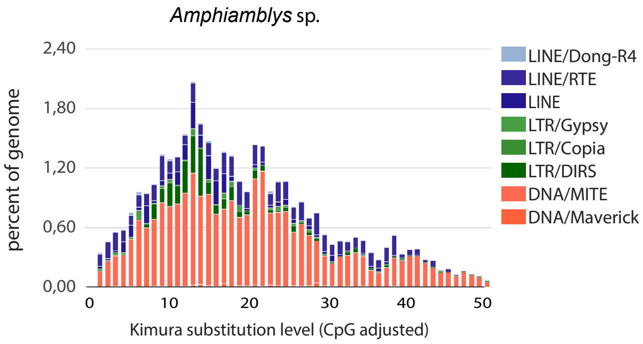
Amphiamblys sp.
percent of genome
2,40
1,80
1,20
0,60
0,00
0
10
20
30
40
50
Kimura substitution level (CpG adjusted)
LINE/Dong-R4
LINE/RTE
LINE
LTR/Gypsy
LTR/Copia
LTR/DIRS
DNA/MITE
DNA/Maverick

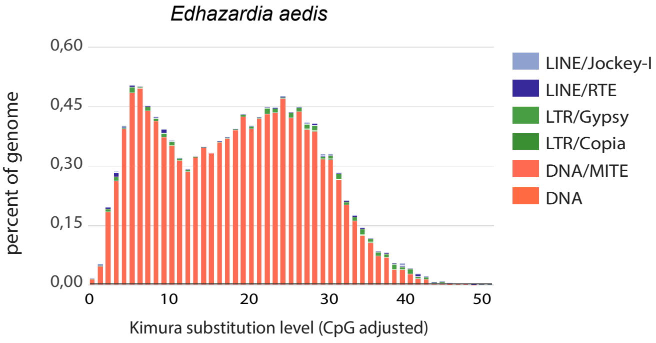
Edhazardia aedis
percent of genome
0,60
0,45
0,30
0,15
0,00
0
10
20
30
40
50
Kimura substitution level (CpG adjusted)
LINE/Jockey-I
LINE/RTE
LTR/Gypsy
LTR/Copia
DNA/MITE
DNA

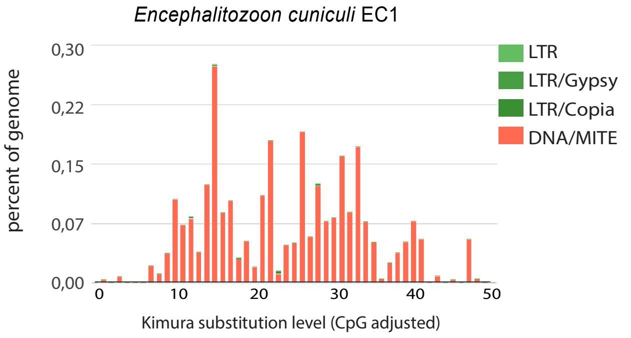
Encephalitozoon cuniculi EC1
percent of genome
0,30
0,22
0,15
0,07
0,00
0
10
20
30
40
50
Kimura substitution level (CpG adjusted)
LTR
LTR/Gypsy
LTR/Copia
DNA/MITE

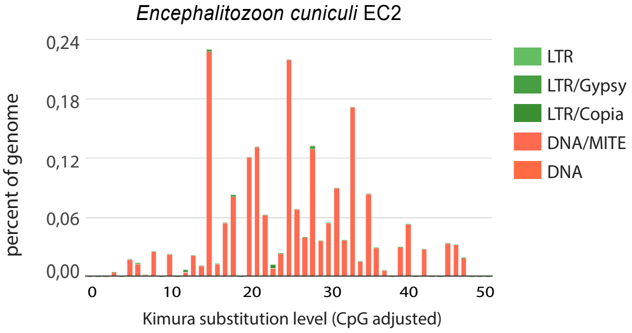
Encephalitozoon cuniculi EC2
percent of genome
0,24
0,18
0,12
0,06
0,00
0
10
20
30
40
50
Kimura substitution level (CpG adjusted)
LTR
LTR/Gypsy
LTR/Copia
DNA/MITE
DNA

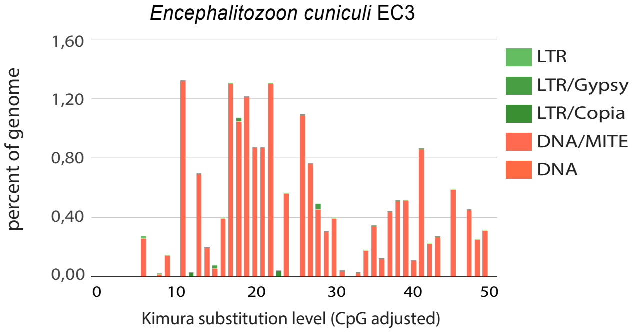
Encephalitozoon cuniculi EC3
percent of genome
1,60
1,20
0,80
0,40
0,00
0
10
20
30
40
50
Kimura substitution level (CpG adjusted)
LTR
LTR/Gypsy
LTR/Copia
DNA/MITE
DNA

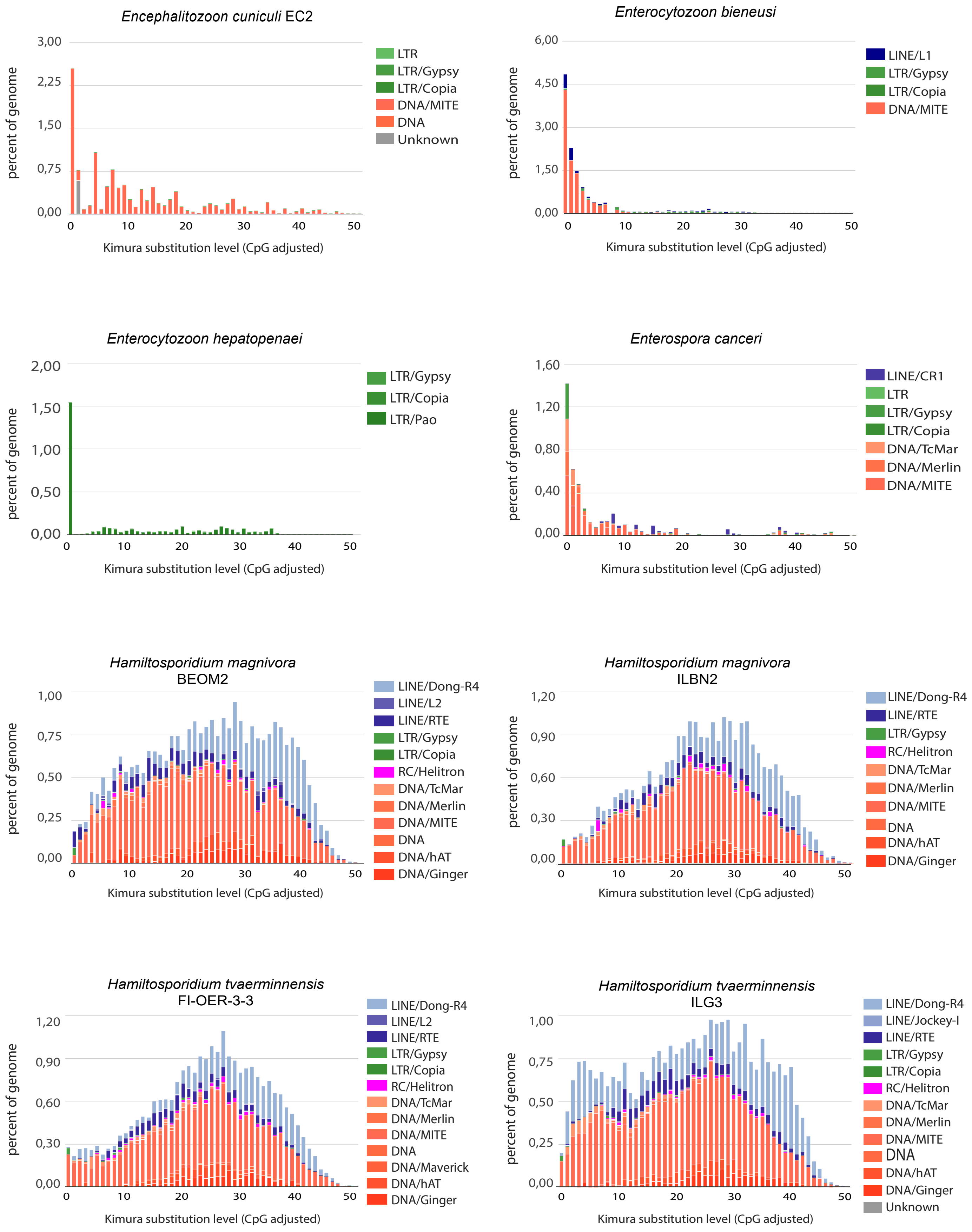
Encephalitozoon cuniculi EC2
percent of genome
3,00
2,25
1,50
0,75
0,00
0
10
20
30
40
50
Kimura substitution level (CpG adjusted)
LTR
LTR/Gypsy
LTR/Copia
DNA/MITE
DNA
Unknown
Enterocytozoon bieneusi
percent of genome
6,00
4,50
3,00
1,50
0,00
0
10
20
30
40
50
Kimura substitution level (CpG adjusted)
LINE/L1
LTR/Gypsy
LTR/Copia
DNA/MITE
Enterocytozoon hepatopenaei
percent of genome
2,00
1,50
1,00
0,50
0,00
0
10
20
30
40
50
Kimura substitution level (CpG adjusted)
LTR/Gypsy
LTR/Copia
LTR/Pao
Enterospora canceri
percent of genome
1,60
1,20
0,80
0,40
0,00
0
10
20
30
40
50
Kimura substitution level (CpG adjusted)
LINE/CR1
LTR
LTR/Gypsy
LTR/Copia
DNA/TcMar
DNA/Merlin
DNA/MITE
Hamiltosporidium magnivora
BEOM2
percent of genome
1,00
0,75
0,50
0,25
0,00
0
10
20
30
40
50
Kimura substitution level (CpG adjusted)
LINE/Dong-R4
LINE/L2
LINE/RTE
LTR/Gypsy
LTR/Copia
RC/Helitron
DNA/TcMar
DNA/Merlin
DNA/MITE
DNA
DNA/hAT
DNA/Ginger
Hamiltosporidium magnivora
ILBN2
percent of genome
1,20
0,90
0,60
0,30
0,00
0
10
20
30
40
50
Kimura substitution level (CpG adjusted)
LINE/Dong-R4
LINE/RTE
LTR/Gypsy
RC/Helitron
DNA/TcMar
DNA/Merlin
DNA/MITE
DNA
DNA/hAT
DNA/Ginger
Hamiltosporidium tvaerminnensis
FI-OER-3-3
percent of genome
1,20
0,90
0,60
0,30
0,00
0
10
20
30
40
50
Kimura substitution level (CpG adjusted)
LINE/Dong-R4
LINE/L2
LINE/RTE
LTR/Gypsy
LTR/Copia
RC/Helitron
DNA/TcMar
DNA/Merlin
DNA/MITE
DNA
DNA/Maverick
DNA/hAT
DNA/Ginger
Hamiltosporidium tvaerminnensis
ILG3
percent of genome
1,00
0,75
0,50
0,25
0,00
0
10
20
30
40
50
Kimura substitution level (CpG adjusted)
LINE/Dong-R4
LINE/Jockey-I
LINE/RTE
LTR/Gypsy
LTR/Copia
RC/Helitron
DNA/TcMar
DNA/Merlin
DNA/MITE
DNA
DNA/hAT
DNA/Ginger
Unknown

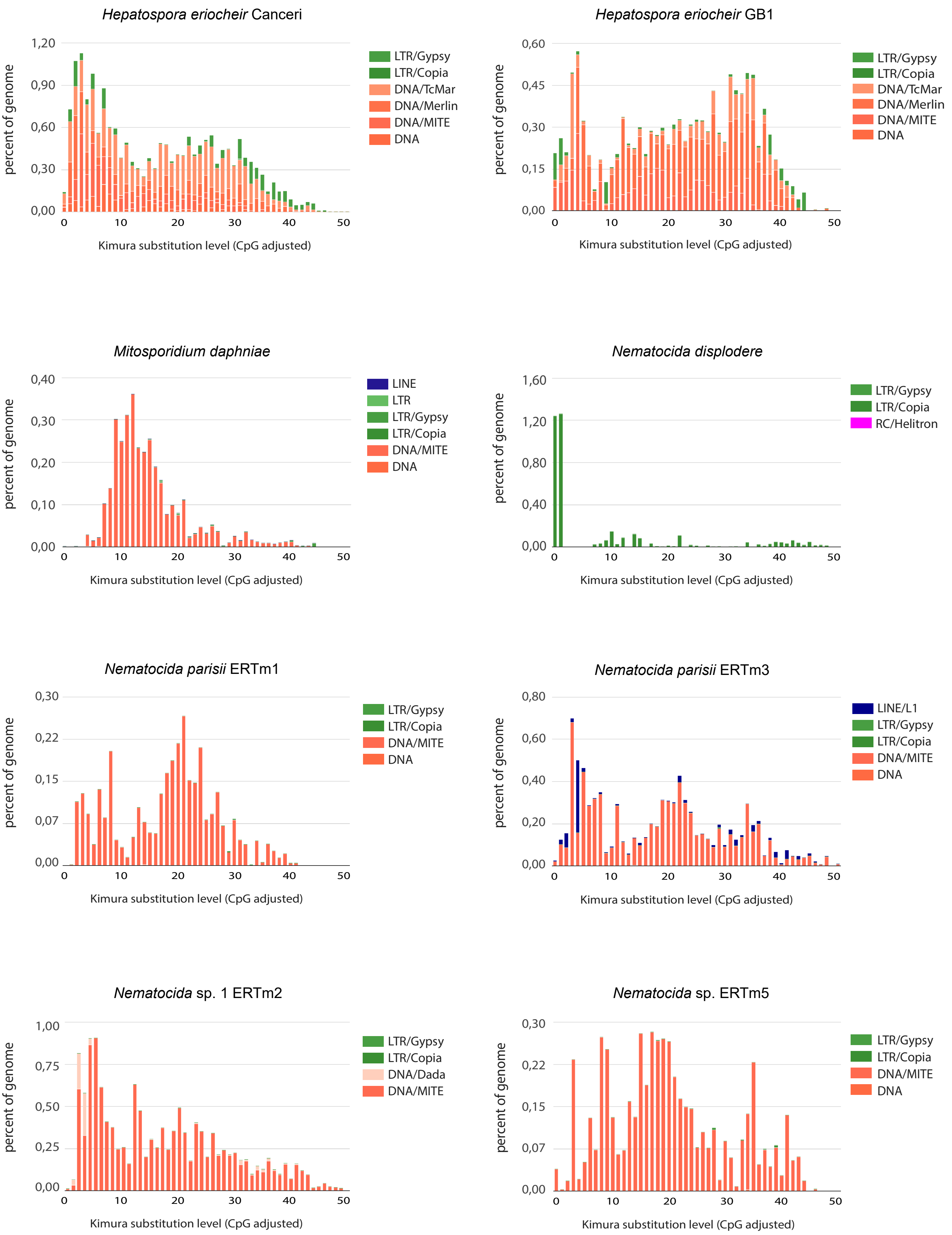

Hepatospora eriocheir Canceri
percent of genome
Kimura substitution level (CpG adjusted)
LTR/Gypsy
LTR/Copia
DNA/TcMar
DNA/Merlin
DNA/MITE
DNA
Hepatospora eriocheir GB1
percent of genome
Kimura substitution level (CpG adjusted)
LTR/Gypsy
LTR/Copia
DNA/TcMar
DNA/Merlin
DNA/MITE
DNA
Mitosporidium daphniae
percent of genome
Kimura substitution level (CpG adjusted)
LINE
LTR
LTR/Gypsy
LTR/Copia
DNA/MITE
DNA
Nematocida displodere
percent of genome
Kimura substitution level (CpG adjusted)
LTR/Gypsy
LTR/Copia
RC/Helitron
Nematocida parisii ERTm1
percent of genome
Kimura substitution level (CpG adjusted)
LTR/Gypsy
LTR/Copia
DNA/MITE
DNA
Nematocida parisii ERTm3
percent of genome
Kimura substitution level (CpG adjusted)
LINE/L1
LTR/Gypsy
LTR/Copia
DNA/MITE
DNA
Nematocida sp. 1 ERTm2
percent of genome
Kimura substitution level (CpG adjusted)
LTR/Gypsy
LTR/Copia
DNA/Dada
DNA/MITE
Nematocida sp. ERTm5
percent of genome
Kimura substitution level (CpG adjusted)
LTR/Gypsy
LTR/Copia
DNA/MITE
DNA

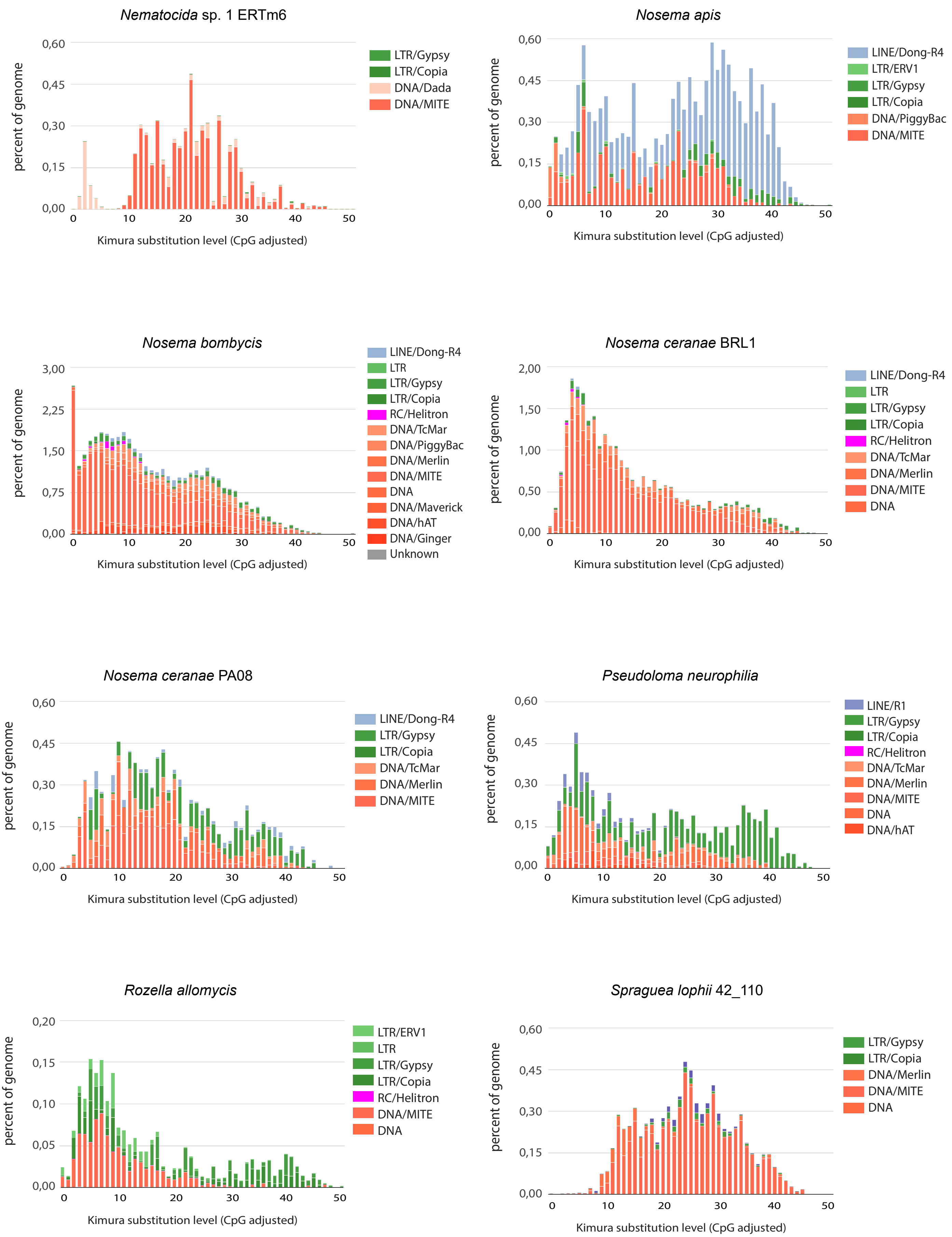

Nematocida sp. 1 ERTm6
percent of genome
0,00
0,15
0,30
0,45
0,60
0
10
20
30
40
50
Kimura substitution level (CpG adjusted)
LTR/Gypsy
LTR/Copia
DNA/Dada
DNA/MITE
Nosema apis
LINE/Dong-R4
LTR/ERV1
LTR/Gypsy
LTR/Copia
DNA/PiggyBac
DNA/MITE
Nosema bombycis
0,75
1,50
2,25
3,00
LINE/Dong-R4
LTR
LTR/Gypsy
LTR/Copia
RC/Helitron
DNA/TcMar
DNA/PiggyBac
DNA/Merlin
DNA/MITE
DNA
DNA/Maverick
DNA/hAT
DNA/Ginger
Unknown
Nosema ceranae BRL1
0,50
1,00
1,50
2,00
LINE/Dong-R4
LTR
LTR/Gypsy
LTR/Copia
RC/Helitron
DNA/TcMar
DNA/Merlin
DNA/MITE
DNA
Nosema ceranae PA08
LINE/Dong-R4
LTR/Gypsy
LTR/Copia
DNA/TcMar
DNA/Merlin
DNA/MITE
Pseudoloma neurophilia
LINE/R1
LTR/Gypsy
LTR/Copia
RC/Helitron
DNA/TcMar
DNA/Merlin
DNA/MITE
DNA
DNA/hAT
Rozella allomycis
0,05
0,10
0,15
0,20
LTR/ERV1
LTR
LTR/Gypsy
LTR/Copia
RC/Helitron
DNA/MITE
DNA
Spraguea lophii 42_110
LTR/Gypsy
LTR/Copia
DNA/Merlin
DNA/MITE
DNA

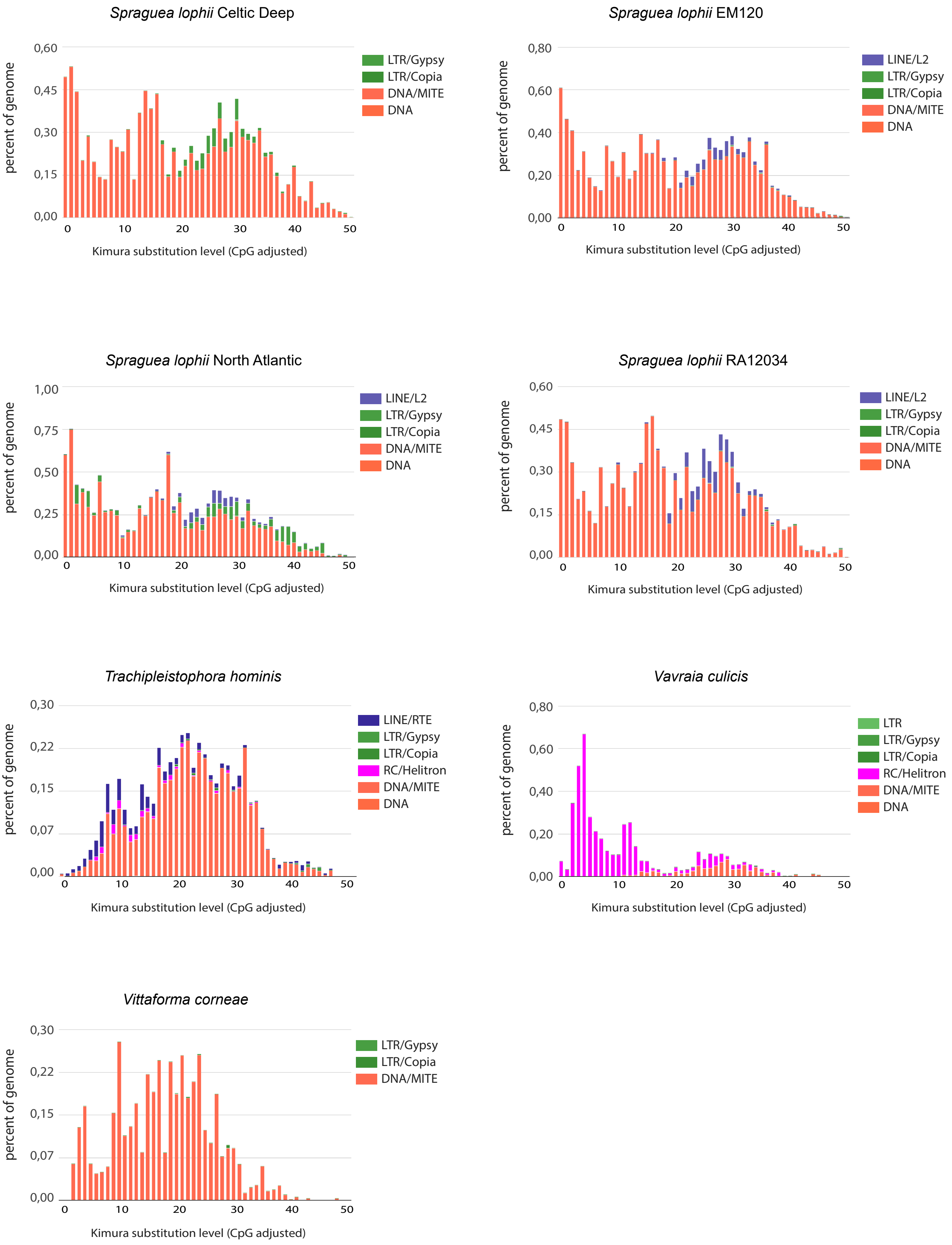

Spraguea lophii Celtic Deep
percent of genome
0,60
0,45
0,30
0,15
0,00
0
10
20
30
40
50
Kimura substitution level (CpG adjusted)
LTR/Gypsy
LTR/Copia
DNA/MITE
DNA
Spraguea lophii EM120
percent of genome
0,80
0,60
0,40
0,20
0,00
0
10
20
30
40
50
Kimura substitution level (CpG adjusted)
LINE/L2
LTR/Gypsy
LTR/Copia
DNA/MITE
DNA
Spraguea lophii North Atlantic
percent of genome
1,00
0,75
0,50
0,25
0,00
0
10
20
30
40
50
Kimura substitution level (CpG adjusted)
LINE/L2
LTR/Gypsy
LTR/Copia
DNA/MITE
DNA
Spraguea lophii RA12034
percent of genome
0,60
0,45
0,30
0,15
0,00
0
10
20
30
40
50
Kimura substitution level (CpG adjusted)
LINE/L2
LTR/Gypsy
LTR/Copia
DNA/MITE
DNA
Trachipleistophora hominis
percent of genome
0,30
0,22
0,15
0,07
0,00
0
10
20
30
40
50
Kimura substitution level (CpG adjusted)
LINE/RTE
LTR/Gypsy
LTR/Copia
RC/Helitron
DNA/MITE
DNA
Vavraia culicis
percent of genome
0,80
0,60
0,40
0,20
0,00
0
10
20
30
40
50
Kimura substitution level (CpG adjusted)
LTR
LTR/Gypsy
LTR/Copia
RC/Helitron
DNA/MITE
DNA
Vittaforma corneae
percent of genome
0,30
0,22
0,15
0,07
0,00
0
10
20
30
40
50
Kimura substitution level (CpG adjusted)
LTR/Gypsy
LTR/Copia
DNA/MITE
