# Supplementary material for: Transposable element abundance correlates with mode of transmission in microsporidian parasites
Source: Mob DNA. 2020 Jun 23;11:19. doi: 10.1186/s13100-020-00218-8 (PMC7313128; doi:10.1186/s13100-020-00218-8)
Supplement: Supplementary file 7 — Additional file 7. Regression analyses of the amount of TEs in relation to genome size with and without phylogenetic independent contrasts. [file 13100_2020_218_MOESM7_ESM.pdf]

Regression analyses

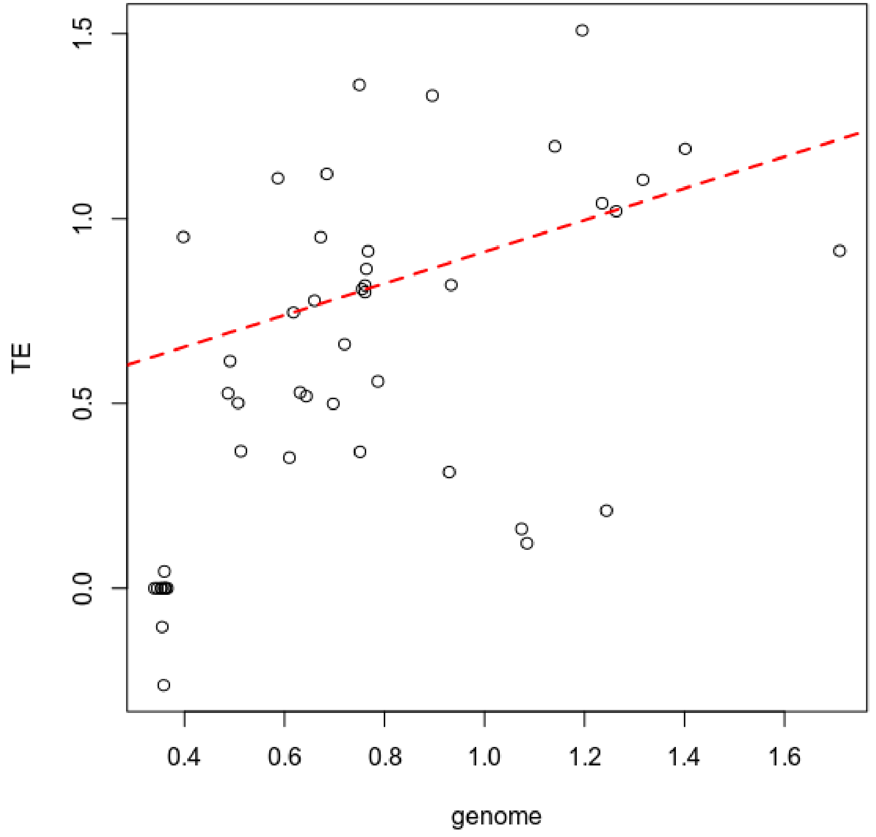

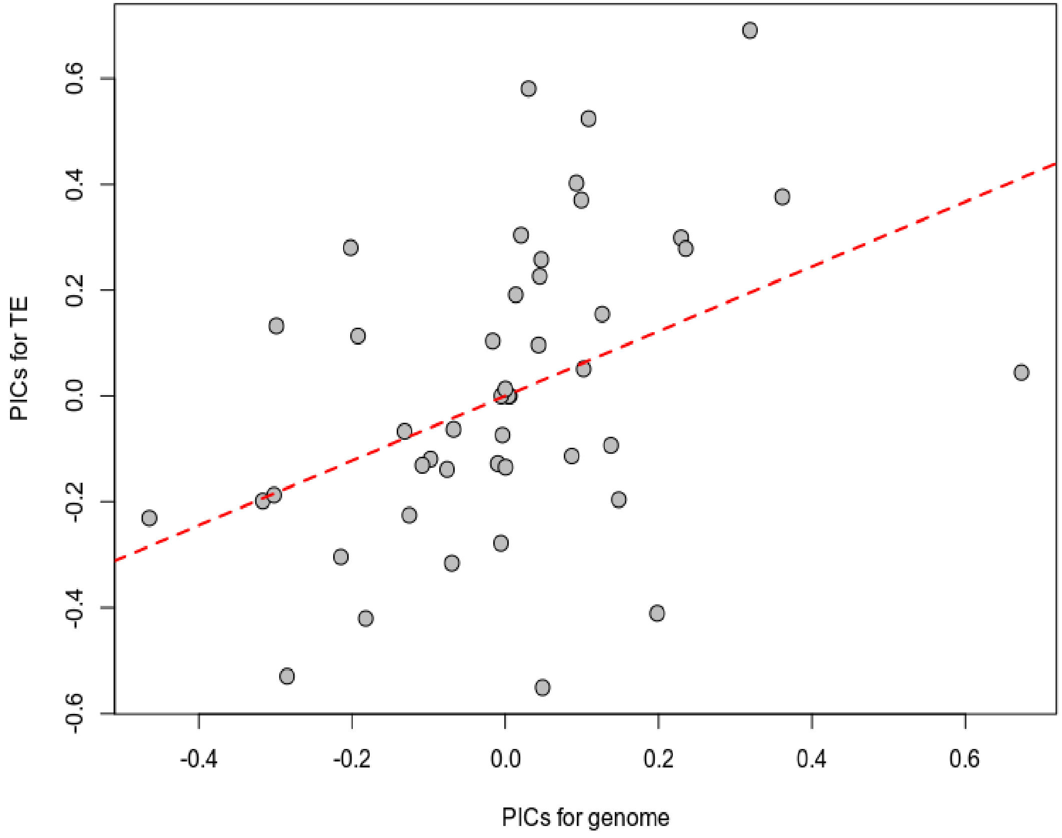

Without contrasts (log transformed data)
Residual standard error: 0.2768 on 45 degrees of freedom
Multiple R-squared: 0.3435, Adjusted R-squared: 0.3289
F-statistic: 23.55 on 1 and 45 DF, p-value: 1.501e-05

With phylogenetic independent contrasts (PIC)
Residual standard error: 0.2574 on 45 degrees of freedom
Multiple R-squared: 0.1802, Adjusted R-squared: 0.1619
F-statistic: 9.889 on 1 and 45 DF, p-value: 0.002944
