# Supplementary material for: Transposable element abundance correlates with mode of transmission in microsporidian parasites
Source: Mob DNA. 2020 Jun 23;11:19. doi: 10.1186/s13100-020-00218-8 (PMC7313128; doi:10.1186/s13100-020-00218-8)

Multiple alignment of representative microsporidian and lepidopteran Argonaute proteins.

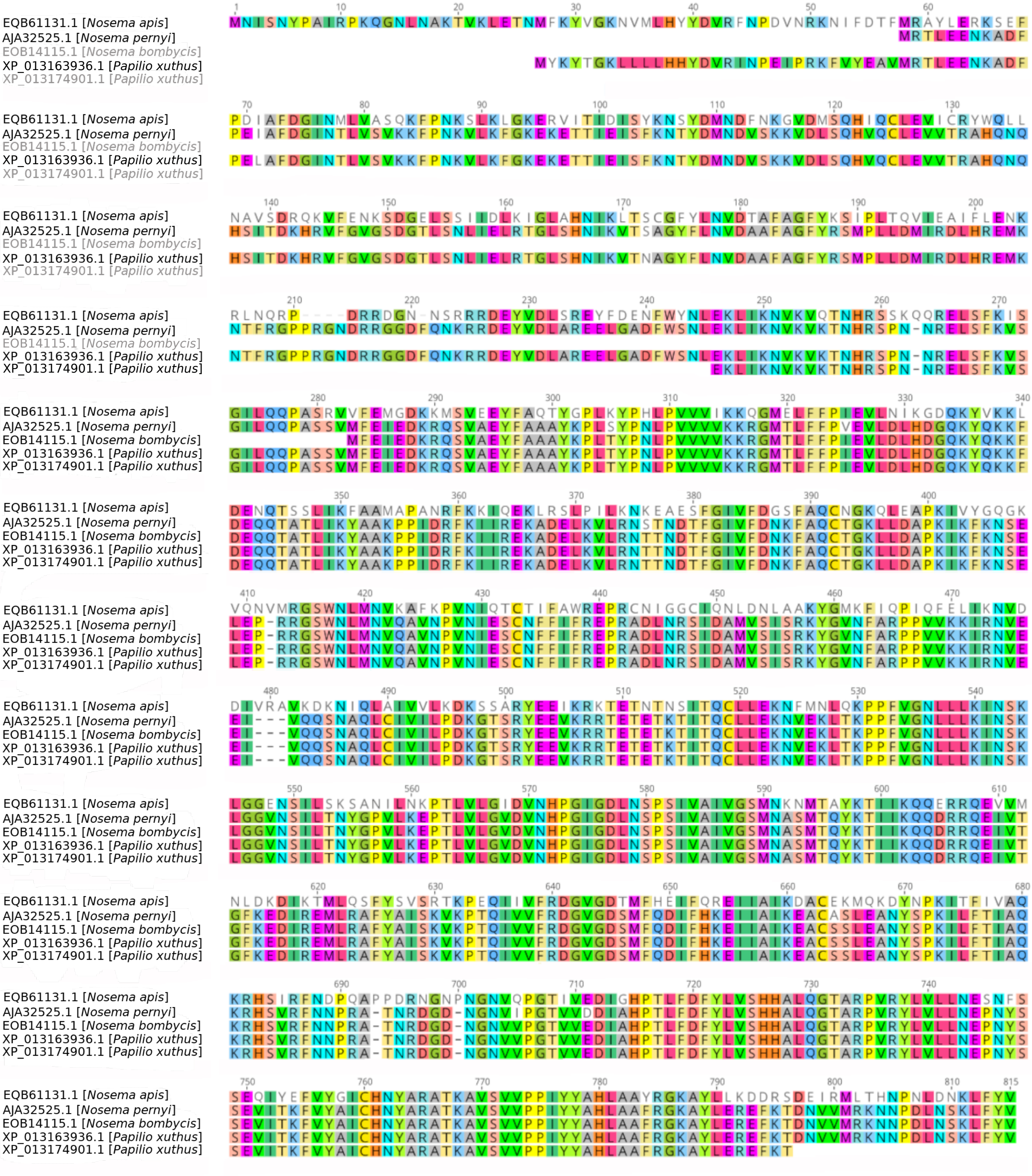

ML phylogeny based on *Argonaute* nucleotide sequences from representative microsporidian (pink) and insect (blue) taxa. The *P. xuthus* genome assembly conains three contigs encoding Argonaute proteins that cluster with microsporidian sequences, suggesting HGT.

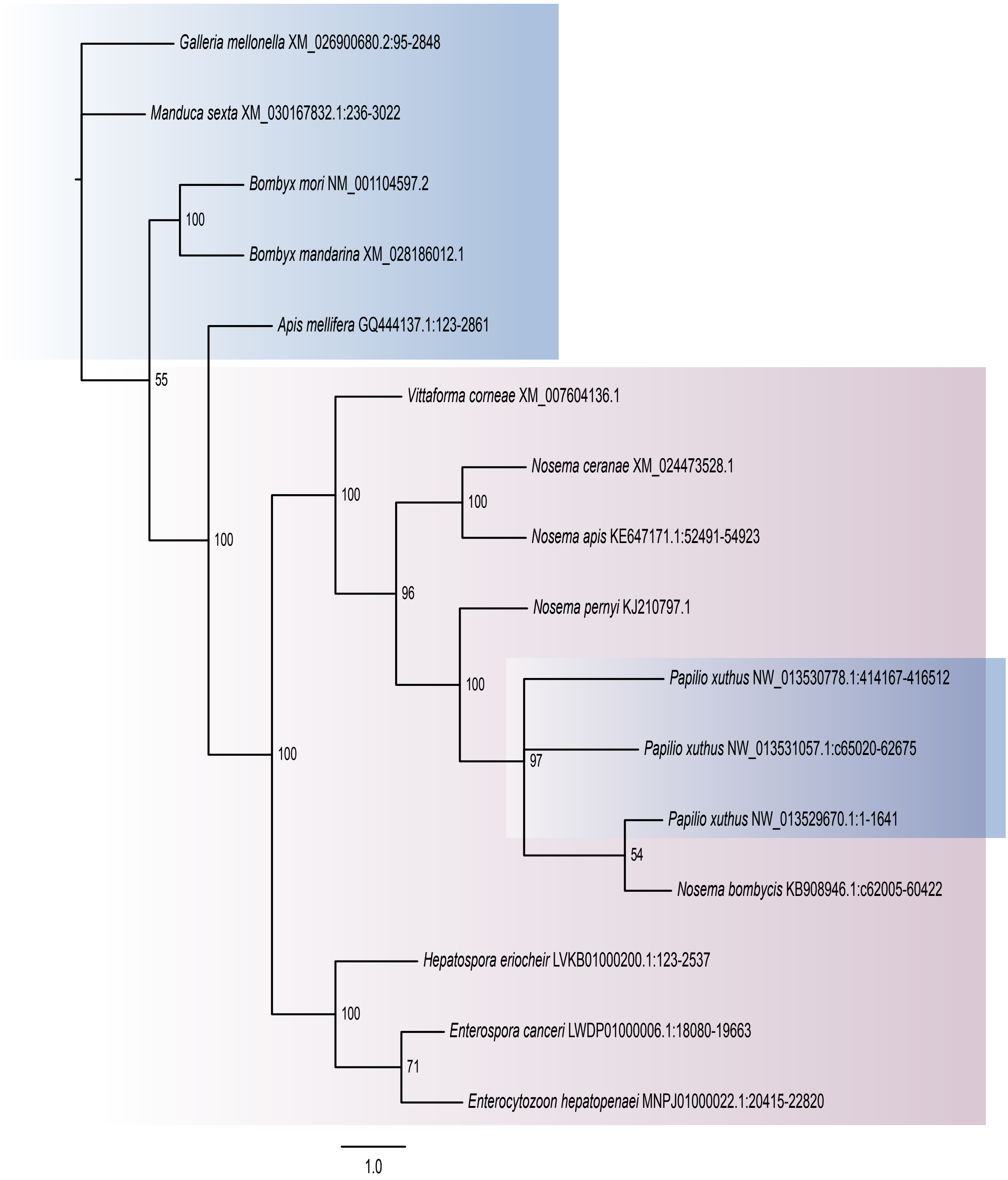

Supplement: Supplementary file 8 — Additional file 8. Protein alignment of representative microsporidian and lepidopteran Argonaute sequences; Argonaute phylogeny testing the direction of the HGT event. [file 13100_2020_218_MOESM8_ESM.pdf]
